# Imputation integrates single-cell and spatial gene expression data to resolve transcriptional networks in barley shoot meristem development

**DOI:** 10.1038/s41477-025-02176-6

**Published:** 2026-01-07

**Authors:** Edgar Demesa-Arevalo, Hannah Dӧrpholz, Isaia Vardanega, Jan Eric Maika, Itzel Pineda-Valentino, Stella Eggels, Tobias Lautwein, Karl Kӧhrer, Thorsten Schnurbusch, Maria von Korff, Bjӧrn Usadel, Rüdiger Simon

**Affiliations:** 1 Institute for Developmental Genetics, Faculty of Mathematics and Natural Sciences, Heinrich-Heine University, Düsseldorf, Germany; 2 Institute for Plant Genetics, Faculty of Mathematics and Natural Sciences, Heinrich-Heine University, Düsseldorf, Germany; 3 Cluster of Excellence on Plant Sciences (CEPLAS), Heinrich-Heine University, Düsseldorf, Germany; 4 Institute of Bio and Geosciences (IBG-4: Bioinformatics) and Bioeconomy Science Center (BioSC), Forschungszentrum Jülich, Jülich, Germany; 5 Institute for Biological Data Science, Faculty of Mathematics and Natural Sciences, Heinrich-Heine University, Düsseldorf, Germany; 6 Genomics and Transcriptomics Laboratory (GTL), Biological and Medical Research Center (BMFZ), Medical Faculty, Heinrich-Heine University, Düsseldorf, Germany; 7 Research Group Plant Architecture, Leibniz Institute of Plant Genetics and Crop Plant Research (IPK), OT Gatersleben, Seeland, Germany; 8 Institute of Agricultural and Nutritional Sciences, Faculty of Natural Sciences III, Martin Luther University Halle-Wittenberg, Halle, Germany; 9 Present Address: Genetic Engineering Department, Irapuato Unit, Center for Research and Advanced Studies of the IPN (Cinvestav), Irapuato, Mexico

**Keywords:** Shoot apical meristem, Cell fate, Plant molecular biology

## Abstract

Grass inflorescences are composite structures, featuring complex sets of meristems as stem cell niches that are initiated in a repetitive manner. Meristems differ in identity and longevity, generate branches or split to form flower meristems that finally produce seeds. Within meristems, distinct cell types are determined by positional information and the regional activity of gene regulatory networks. Understanding these local microenvironments requires precise spatio-temporal information on gene expression profiles, which current technology cannot achieve.

Here we investigate transcriptional changes during barley development, from the specification of meristem and organ founder cells to the initiation of distinct floral organs, on the basis of an imputation approach integrating deep single-cell RNA sequencing with spatial gene expression data. The expression profiles of more than 40,000 genes can now be analysed at cellular resolution in multiple barley tissues using the new web-based graphical interface BARVISTA, which enables precise virtual microdissection to analyse any sub-ensemble of cells. Our study pinpoints previously inaccessible key transcriptional events in founder cells during primordia initiation and specification, characterizes complex branching mutant phenotypes by barcoding gene expression profiles, and defines spatio-temporal trajectories during flower development. We thus uncover the genetic basis of complex developmental processes, providing novel opportunities for precisely targeted manipulation of barley traits.

## Abstract (web summary)

Spatially resolved gene expression during barley development was done by integrating an scRNA-seq dataset from cells with unknown position with spatial transcriptomics. This dataset is publicly available through the online web-based BARVISTA application.

## Main

Plant development depends on the activities of stem cell systems, the meristems, that give rise to organ primordia or new meristems in distinct patterns. In the grass family, flower development involves the sequential specification of meristematic identities at the inflorescence meristem, leading to diverse architectures^1,2^. Rice and oat plants develop multiple branched panicles, whereas teosinte, rye, wheat and barley form simpler spikes that generate rows of floret meristems, probably reflecting an ancestral state^3^. There is a strong genetic basis for these inflorescence architectures which can be explored by studying mutants or genetic variation affecting meristem behaviour during domestication. For example, the evolution of maize from teosinte involved the repression of axillary meristems (AMs), enlargement of the inflorescence meristem (IM), and a switch from distichous to spiral inflorescence phyllotaxis, forming multiple kernels at each node^4^.

In barley, a spike-type inflorescence axis (rachis) generates an AM at each node in a distichous pattern subtended by a developmentally repressed leaf meristem. AMs differentiate into triple spikelet meristems (TSMs) that separate to form three distinct spikelet meristems (SMs), two lateral (LSM) and one central (CSM)^5^. Each spikelet initiates a determinate meristem that forms a short axis (rachilla), subtending bracts and a single floret meristem (FM) that generates the floral organs. When the indeterminate IM at the tip of the rachis has generated a finite number of TSMs, the IM and several SMs undergo gradual degeneration in a basipetal sequence, indicating that position along the rachis is a key fate determinant. Each CSM forms a fertile flower and a single grain, whereas LSMs develop to fertility only in six-rowed barley varieties, but remain small and sterile in two-rowed barley. Analysis of barley mutants uncovered that LSM fertility and the determinacy of TSMs is regulated by the LOB-domain transcription factor (TF) *VRS4* (*Hordeum vulgare RAMOSA2*, or *HvRA2*) and the TCP-family TF *INT-C*^6,7^. Loss-of-function mutations in the *INT-M* gene, encoding an AP2-like TF, or in *COMPOSITUM1 (COM1)*, encoding a TB1/CYC/PCF (TCP)-like TF whose expression at the IM–SM boundary depends on *HvRA2*, cause the rachilla to give rise to a new branch or generate more florets per spikelet^8,9^, indicating that *COM1* promotes meristem determinacy. Mutations in *HvCLV1*, encoding a CLAVATA1 family receptor kinase, or in *HvFCP1*, encoding a secreted signalling peptide that acts through *HvCLV1*, enhance rachilla indeterminacy and fail to maintain *COM2* expression and AM initiation at the IM^10^. At higher temperatures, rachilla determinacy is promoted by the MADS-box transcription factor (MADS TF) HvMADS33/HvMADS1, a member of the *LOFSEP* clade associated with inflorescence architecture in rice and several dicot species^11^.

The homeodomain TF *KNOTTED1* (*KN1*), first described in maize, promotes meristem indeterminacy during vegetative and reproductive development and is expressed in vegetative shoot apical meristems (vSAM) and IMs, but not in organ founder cells^12^. Lateral meristems later regain *KN1* expression. *KN1* RNA is mostly absent from the outermost cell layer (the L1) of meristems, but KN1 protein together with *KN1*-mRNA can enter the L1 via plasmodesmata^13–15^. Local auxin accumulation and absence of *KN1* expression mark organ initiation in meristems of many species^16^. However, gene regulatory networks (GRNs) responsible for primordia specification, their identities, determinacy and fates are still unknown^1,2,8,17^. To explore GRNs underlying meristem specification and organ initiation in an unbiased manner, we generated single-cell RNA-sequencing (scRNA-seq) data for the barley vSAM and inflorescence (spike). Several scRNA-seq datasets are available in grass species^18–31^, and deduced developmental trajectories can illustrate shifts in cell fate and gene expression patterns. However, the origin and fate of cells within complex tissues can only be inferred indirectly from the known expression profiles of prominent marker genes^32^. We could precisely localize and quantify transcripts of 86 genes on tissue sections at cellular resolution using the Molecular Cartography (MC) platform for multiplexed single-molecule RNA fluorescence in situ hybridization (smRNA-FISH). We integrated the scRNA-seq and smRNA-FISH data to generate imputed transcriptome-wide single-cell expression matrices. The combined datasets are presented in the user-friendly, searchable online database BARVISTA (barley virtual in situ transcriptome atlas), developed in-house as part of this study, which visualizes the expression of 48,904 barley genes at cellular resolution on tissue sections representing different developmental stages. BARVISTA enables the virtual microdissection of cell populations from these sections, followed by reclustering and mining for regionally specific gene expression profiles. We used BARVISTA to show that *HvKN1* transcripts, which are absent from most meristem L1 cells^14^, can be detected in a subpopulation of L1 cells at the tip of incipient primordia, where *HvKN1* is co-expressed with cytokinin biosynthesis genes, which could establish a positive feedback loop that promotes meristem indeterminacy. We observed rapid changes in gene expression complexity during SM development, revealing expression fingerprints that allowed us to phenotype the complex cell identities of meristem mutants. Our integrated dataset revealed gene expression dynamics with unprecedented spatio-temporal resolution, providing a framework for the formation and specification of meristems and organ primordia during barley development.

## A comprehensive single-cell gene expression atlas of barley meristems

**Fig. 1:**
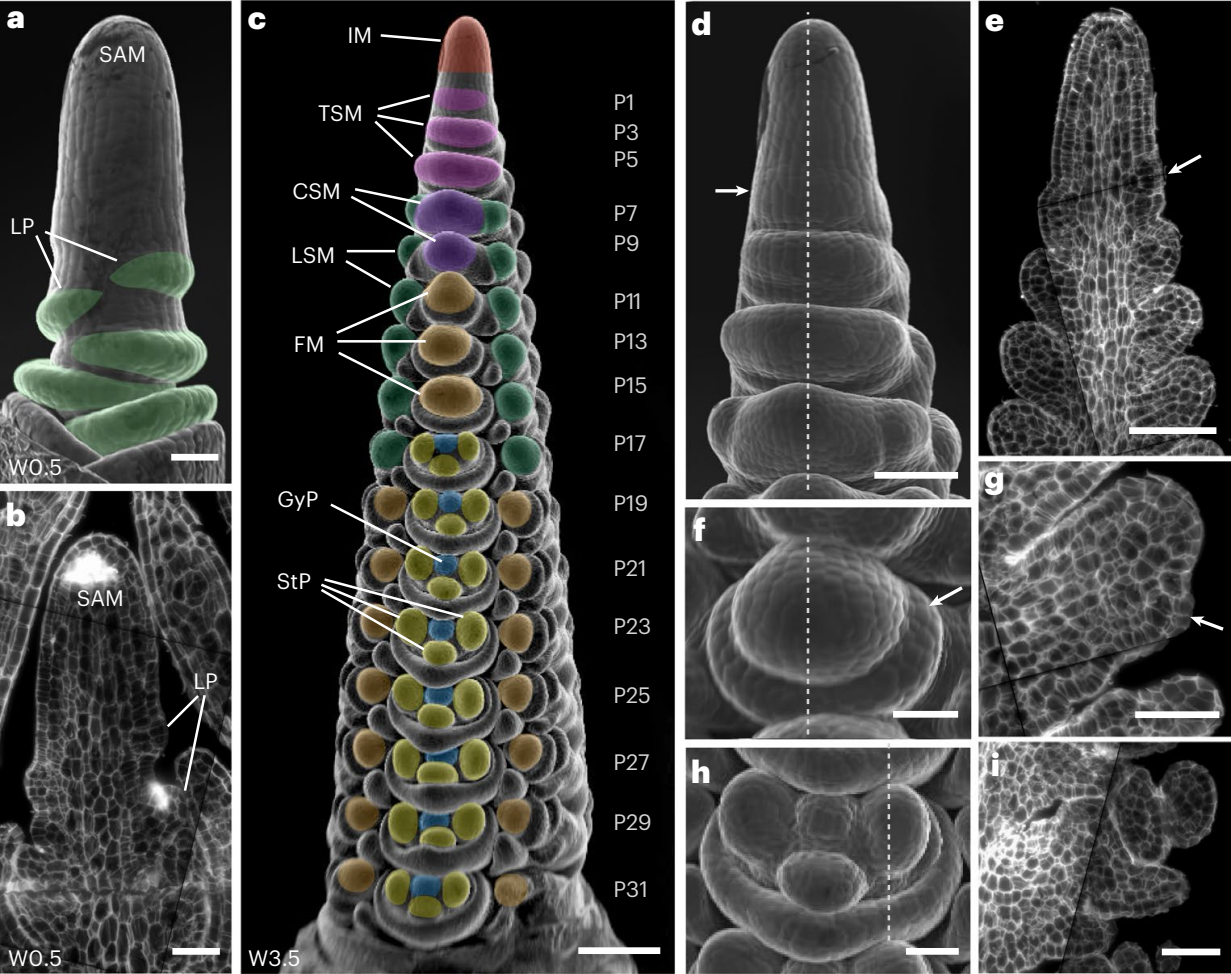
Organ formation in developing spikes of barley. **a**, Scanning electron micrograph (SEM) of the vegetative shoot apical meristem (vSAM), highlighting leaf primordium (LP) formation (green). **b**, Sagittal section of the vSAM. **c**, At W3.5, the inflorescence meristem (IM) at the top of the spike is determined to form lateral meristems specified as triple spikelet meristems (TSMs), which will then generate two lateral spikelet meristems (LSMs) and one central spikelet meristem (CSM). In the Golden Promise genetic background, a two-row type of barley, only the CSM continues its development and will form a floret meristem (FM), which then generates gynoecium (GyP) and stamen primordia (StP). Plastochrons are indicated as P1–P31. **d**, SEM of the top part of a developing spike, where the TSM is specified and apparent as a protrusion on the surface axillary to the IM (arrow). **e**, A sagittal section through the IM and TSM. The earliest sign of the TSM is cell proliferation where the primordium is formed (arrow). **f**, SEM of an FM with lemma primordium (arrow). **g**, FM sagittal section showing the outgrowing lemma (arrow). **h**, SEM of one floret developing StP and GyP. **i**, Sagittal section through one floret. Dotted lines in **d**, **f** and **h** are equivalent in position to the sagittal sections in **e**, **g** and **i**, respectively. SEM images are representative of their respective developmental stage. Scale bars, 200 µm (**c**); 100 µm (**d**,**e**); 50 µm (**a**,**b**,**f**–**i**).

**Fig. 2:**
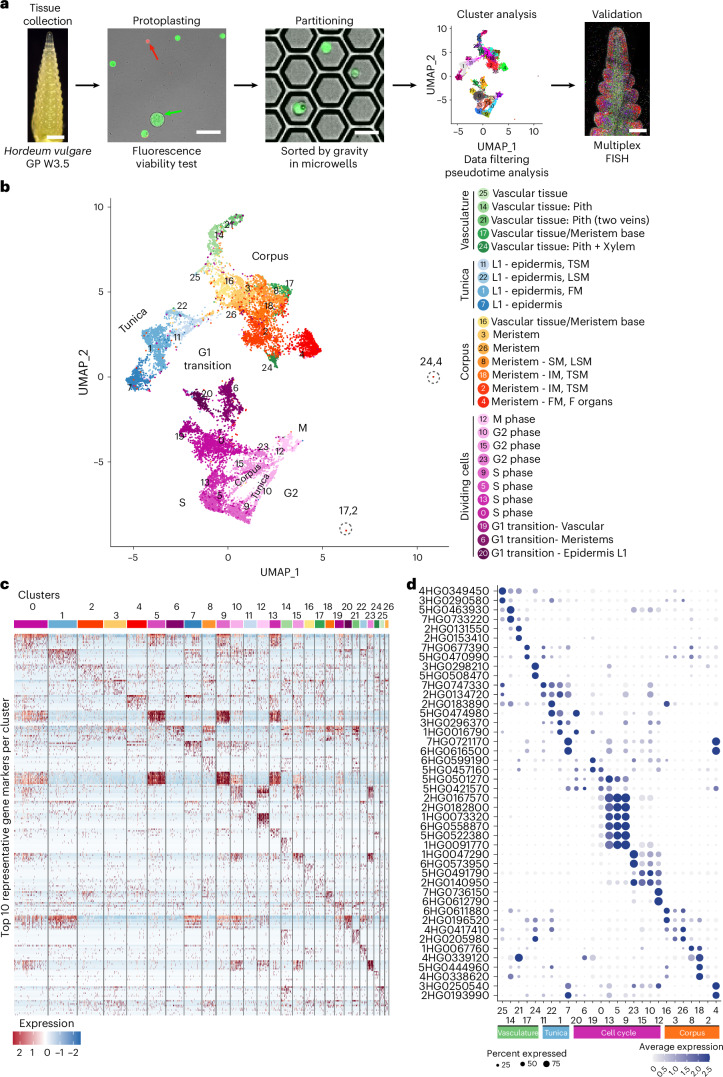
A single-cell atlas from developing barley spikes. **a**, Workflow for the isolation, sequencing, analysis and validation of protoplast scRNA-seq from developing barley spikes at W3.5. **b**, UMAP for dimension reduction revealed that the cluster identities are organized as four populations containing vasculature, tunica, corpus and dividing cells (abbreviations as in Fig. 1). **c**, Heat map of 10 representative marker genes per cluster. **d**, Expression of two representative marker genes organized by cell populations. Scale bars, 200 and 50 µm (**a**).

Development of the barley SAM and spike can be described using the Waddington scale (Waddington stages, W)^33^. From W0 to W1, the vSAM forms lateral leaf primordia in a distichous pattern, which ensheathe the base of the vSAM (Fig. 1a,b). The vSAM then elongates and transitions to the reproductive stage (IM), characterized by the formation of SMs instead of leaf primordia. By W3.5, the spike has generated two rows of spikelets with a complex developmental gradient. TSMs form from small protrusions on the IM lateral surface (Fig. 1c–e). At P7, the TSM has separated into the CSM and two LSMs. Towards the base, P11–P15 are floral meristems (FM) subtended by lemma primordia. P17–P31 carry one carpel and three stamen primordia, but formation and differentiation of further floral organs is delayed (Fig. 1f–i). To identify transcriptional changes underpinning formation and differentiation of IM, TSM, CSM, LSM and FM, we generated scRNA-seq data from vSAMs at W0.5 and spikes at W3.5 for the barley reference cultivar Golden Promise (GP). Stage W3.5 captures gene expression information for all meristem types that finally contribute to flower organs (Fig. 2a). Enzymatic digestion of meristem cell walls and release of viable protoplasts from meristems can be hindered by differences in cell wall composition compared to leaf cells^34^. We assessed the ability to capture the full diversity of spike cells and tissues by representation of cell/protoplast sizes, comparing intact with protoplasted tissues. We also used the reporter line *pHvFCP1:mVenus-H2B*, which expresses Venus in specific meristematic tissues including the stem cells^10^ (Supplementary Fig. 1a,b,h). Protoplast viability was monitored by differential staining with calcein-acetoxymethyl ester, which fluoresces in the cytoplasm of viable cells following uptake and esterase cleavage, and DRAQ7, which stains nuclei only in membrane-compromised or dead cells^35^. Samples with more than 65% viable protoplasts were loaded for microwell-based cell isolation. Cells from inflorescence spikes and vSAMs were isolated in three independent experiments, and we obtained high-quality transcriptome information from 16,528 individual cells for W3.5 (ref. ^36^). We detected the expression of an average of 4,527 genes per cell and 17,553 transcripts, mapping to 46,495 gene models, including low- and high-confidence models, of the reference genome assembly for barley cultivar Morex (Morex V3). Additional libraries from wild-type (WT) vSAM and *com1a;com2g* mutant spikes recovered data from 919 and 5,196 cells, respectively (Fig. 2b and Supplementary Table 1). Comparing bulk RNA-seq data from intact and protoplasted tissues identified 3,684 genes that were differentially expressed in response to protoplasting (Supplementary Fig. 1c–g and Table 2). Removing the protoplasting-induced differentially expressed genes (DEGs) in bulk RNA-seq increased the positive correlation between transcriptomes coming from protoplast versus intact meristems. We then subtracted the protoplasting-induced DEGs and integrated the scRNA-seq data for the three independent experiments using the most variable 7,500 genes, followed by dimensional reduction for clustering analysis, reducing the noise due to protoplasting. We identified 27 clusters with 4,256 putative unique markers genes (Fig. 2a–d and Supplementary Table 3). Cluster ontogenies were determined using known marker genes (Supplementary Table 4).

## scRNA-seq identifies markers specific for tissue lineages

We identified four distinct populations of cells: vasculature, tunica, corpus and dividing cells (Fig. 2b–d and Supplementary Fig. 2). Each population comprised multiple clusters, separated by groups of cells potentially derived from specific meristem types. We first used *HvKN1* (4HG0339120) to identify clusters of meristematic and vascular tissues. A complementary group of clusters representing the tunica expressed *HvHDZIV8* (2HG0198150), which encodes a homeodomain-leucine zipper TF related to maize *OUTER CELL LAYER* and the *Arabidopsis* L1 marker *MERISTEM LAYER1* (*AtML1*)^37^. Additional marker genes identified specific subpopulations in the tunica and corpus: for the IM and TSM, *HvMADS42*/*MADS34* (5HG0511250); for the lemma and CSM, *HvMADS33*/*MADS1* (4HG0396400); for the CSM and floret organs, *HvMADS68*/*MADS7* (7HG0684020); for TSM founder cells, glumes and LSM, *VRS4* (3HG0233930); for the floret meristem, *HvMADS50*/*MADS6* (6HG0604360); and for stamen primordia, *HvMADS24*/*MADS2* (3HG0307160) and *HvMADS76*/*MADS16* (7HG0721170). Similarly, markers were identified for the vascular tissues: cambium, *HvPXY* (7HG0701280); xylem, *HvVND1* (4HG0410880); phloem, *HvSMXL3* (2HG0167230); and rachis, *HvPSAN* (2HG0113750)^38^. For the cell cycle, we identified S-phase histone markers, *H3* (7HG0658120), *H4* (6HG0550690), *H2A* (5HG0518610) and *H2B* (1HG0067590); G2-phase markers *Cyclin-A* (3HG0249410) and *CDKB* (7HG0680610); and M-phase markers *HvTANGLED1* (1HG0073890), closely related to maize *TANGLED1* encoding a microtubule-binding protein associated with mitotic division^39^; *EARLY NODULIN-LIKE PROTEIN* (1HG0082450) and eisosome protein (4HG0411400) (Supplementary Fig. 2 and Table 4). Our diverse dataset ranged from cell-lineage initials to differentiated cells. On the basis of 7,500 variable genes, we captured cell-cycle specific transcriptomes for the tunica and corpus, as well as phase-specific markers. To avoid biases due to cell-cycle phases, we then focused on cells in G1.

## Combination of smRNA-FISH and scRNA-seq identifies meristem subdomains

**Fig. 3:**
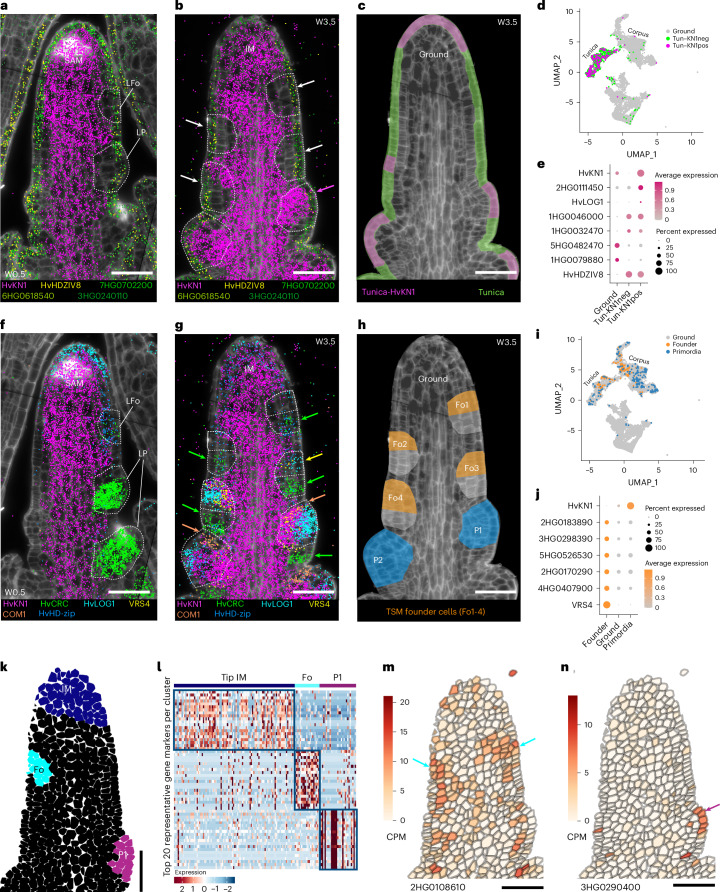
Identification of genetic determinants in primordia and early meristem specification. **a**, In vSAM, the formation of leaf primordia (LP) is preceded by the depletion of *HvKN1* mRNA (magenta dots) in the leaf founder cells (LFo) and during LP growth. **b**, In inflorescence meristems (IM), spikelet founder cells (white arrows and dotted line) also show *HvKN1* mRNA depletion, but this is restored once the primordia are organized (magenta arrow). **c**, The organization of tunica and corpus in the IM is reflected by their expression profiles, but in developing spikes we see an overlap of *HvKN1* in the tunica, especially in meristem tips. **d**, Differentially expressed genes in tunica cells expressing *HvKN1*. **e**, Candidate marker genes in tunica cells expressing *HvKN1*. **f**, In the SAM, *HvHD-zip* and *HvCRC* are expressed in the leaf primordia founder cells (LFo), but in the established LP only *HvCRC* remains strongly expressed with no restoration of *HvKN1* in the primordia. **g**, In spikelet founder primordia, where *HvKN1* mRNA is depleted, the suppressed bract is pre-patterned (green arrows) and expressing leaf-like marker genes such as *HvCRC*, and the spikelet meristem founder cells (Fo) can be identified by *VRS4* and *HvLOG1* expression (yellow arrow). One of the earliest signs of triple spikelet meristem primordia is the expression of *HvCOM1* (orange arrows). **h**, Schematic representation showing the spikelet meristem Fo and the triple spikelet meristem primordia (P) in a developing spike of barley. **i**, Founder and primordial cells can be subtracted from scRNA-seq populations using their expression profiles. **j**, Candidate genes differentially expressed in triple spikelet meristem founder cells. **k**, By data integration and imputed gene expression, we can virtually dissect the IM (dark blue), Fo (cyan) and P cells (purple). **l**, Heat map of the top 20 genes expressed differently in each group of cells. Heat maps show selected examples of imputed differentially expressed genes using a one-sided MAST, with *P*_adj_ < 0.05 and with an enrichment of cells expressing the genes in the analysed population more than double the level in the remaining cells (pct.1/pct.2 ≥ 2). **m**, Imputed expression of candidate genes specifically expressed in the founder cells or **n**, in TSM primordium. smRNA-FISH sections are representative of at least 5 independent developing spikes. Scale bars, 50 µm.

We validated cluster annotations by smRNA-FISH^40^ (Supplementary Table 5 and Supplementary Fig. 3). To simplify MADS-box nomenclature, we use the most recent barley annotation (HvMADS)^41^, and genes previously named by homology to characterized rice genes are shown with both names (MADS, Supplementary Table 6). We selected 100 genes from the scRNA-seq data with known or predicted expression patterns based on studies from barley and other organisms (3–4 per cluster) to generate multiplex smRNA-FISH probes. We detected transcripts from 86 genes tested, with a spatial resolution of <140 nm. For example, *HvHDZIV8*, two *HDZIV*-related TFs (7HG0702200 and 6HG0618540) and a pleiotropic drug resistance ABC transporter gene (3HG0240110) were specifically expressed in the tunica of the vSAM (Fig. 3a and Supplementary Table 7) and IM at stage W3.5 (Fig. 3b), whereas *HvKN1* transcripts located to corpus cells. To determine whether the smRNA-FISH data reflected overall expression levels, we compared the number of RNA-seq reads with the number of spots detected by smRNA-FISH, and found a Pearson correlation of 0.73 for genes with a wide range of expression levels, indicating that spot number in smRNA-FISH can serve as a reliable proxy for expression levels (Supplementary Fig. 4a). Following cell segmentation, we assigned counts per gene and per cell and generated an expression matrix for the 86 genes detected across 22,082 segmented cells, with a dynamic range of up to 536 transcripts of a single gene per cell, a maximum of 627 total transcripts per cell and a mean of 95.35 transcripts per cell (Supplementary Fig. 4b–e and Table 5).

## Integrating scRNA-seq with smRNA-FISH data for gene expression imputation

A cell’s position and its gene expression profile inform on developmental time and progression, and the clonal origin of a cell. Current technologies for quantitative spatial gene expression analysis are limited by probe number, sensitivity in molecularly crowded environments and retention or transferability of RNA to capture devices^40,42^. We therefore used the spatially resolved quantitative data as anchors and mapped each cell in the scRNA-seq dataset to its nearest neighbour in the MC dataset. Of the 86 genes in the MC dataset, 81 were expressed in a sufficient number of cells and at differing levels to be regarded as informative. Integration of the datasets in Seurat^43^ following the normalization of gene expression values yielded clusters whose numbers depended on the clustering resolution (cr) parameter, with settings of 0.1 to 2.0. Biologically relevant clusters representing ‘dividing cells’, ‘floret meristem’, ‘xylem’ and ‘L1 – epidermis’ were identified at cr0.3, indicating that these involve highly distinct gene expression patterns, separating them from other cell populations (Supplementary Fig. 5). ‘L1 – FM epidermis’, ‘L1 – F organ epidermis’ and ‘early TSM’ became distinct only beyond cr1.1, indicating fewer differences in gene expression relative to other cell populations.

Spatially resolved data and scRNA-seq data can be aligned using published tools such as TANGRAM; however, these rely on large numbers of shared variable genes across datasets^44^. We therefore developed a custom data imputation approach to map gene expression values from the scRNA-seq data onto MC cells, which operates reliably with a much smaller gene set. In the initial cell similarity determination step, proximate cells from the scRNA-seq experiment were determined for each MC cell using cosine similarity (CS)^45^ (Supplementary Fig. 6a). Gene expression values for each MC cell were then calculated as a weighted average from the 25 most similar scRNA-seq cells, using the CS as weight to ensure that most similar cells had the greatest influence on the calculated average (Supplementary Fig. 6b). Expression levels were imputed for 48,904 genes in the inflorescence sections and 27,656 genes in the vSAM. To validate the method, we compared imputed and experimentally determined gene expression values (Supplementary Fig. 6c) on a cell-by-cell basis for all 22,082 segmented cells, using expression data for the anchor genes to determine CS. A CS of +1 indicates that the entire gene expression profile of a cell can be computationally reproduced. Mean CS values were 0.84 ± 0.11 for the inflorescence sections and 0.78 ± 0.11 for the vSAM, showing that gene expression patterns in most cells could be reconstructed with only minor differences compared to measured values (Supplementary Fig. 7). Because the 81 anchor genes were used for both initial cell similarity determination and validation, we performed simulations to validate the quality of imputation. The scRNA-seq data were split into a test set, for which gene expression was imputed (replacing the MC dataset in the workflow described above), and a reference dataset from which gene expression data were taken. For both imputations, the 81 anchor genes were used during the cell similarity determination step. For the first imputation, validation involved the same 81 genes (scSub IM 81 genes) (Supplementary Fig. 8). For the second validation, all 48,904 genes (scSub IM 48,904 genes) were used for the cell-by-cell comparison of imputed vs experimentally measured values. Validating with the same 81 anchor genes gave a mean CS of 0.84 ± 0.10, and validation with all 48,904 genes gave a still robust, although slightly lower CS of 0.73 ± 0.10 (Supplementary Table 8). We then explored the impact of cell number and gene selection on the accuracy of imputation. Accuracy improved linearly with increasing reference cell number, but plateaued at ~2,500 cells (Supplementary Fig. 9). We found that genes with substantial variations in expression across samples performed better than less variably expressed genes. Genes selected using PERSIST, which identifies representative gene sets for distinct cell populations^46^, outperformed hand-selected or randomly selected genes, and 20 genes were sufficient for reliable cell similarity determination.

Results are available through the new online gene expression atlas BARVISTA, which displays individual cell clusters, uniform manifold approximation and projection (UMAP) plots and imputed gene expression data for all cells in a tissue section. Individual cells or groups can be selected to extract gene expression data for further analysis.

## Exploring gene expression programs during primordia initiation

We used our integrated datasets in BARVISTA to probe gene expression patterns in detail during early primordia initiation. *KN1* is often used as a marker for the meristem corpus, which is mostly absent in the L1 layer and downregulated at sites of organ initiation^14^. Interestingly, maize scRNA-seq data identified some tunica cells expressing *ZmKN1* (ref. ^31^), and the rice orthologue of *ZmKN1* (*OsH1)* is expressed in the L1 of FM, in some cells of the IM, but not in L1 cells of the vSAM^47^. The function and origin of these *KN1* RNAs, which might bind to mobile KN1 protein in the corpus for co-transport to the tunica, is unknown. Expression of *HvKN1* and *HvHDZIV8* identified the tunica cells in our scRNA-seq data (Fig. 3a,b). In the spike, almost 9% (268 of 3,023) of all tunica cells expressed *HvKN1* (Fig. 3c,d), and also the cytokinin biosynthesis gene *HvLOG1* (5HG0502720), as well as *HvWOX9C.2* (3HG0278560) and *HvWOX9C.1* (1HG0088440), which are *WUSCHEL-LIKE HOMEOBOX* genes related to rice *OsWOX9C*. *HvLOG1* is expressed at the tips of the SAM, IM, TSM and FM (Fig. 3e and Supplementary Table 9) and could potentially generate a cytokinin source at meristem apices that promotes *WOX9C* expression. In the vSAM (Supplementary Fig. 10a–d and Table 10), ~12% (17 of 138) of all tunica cells expressed *HvKN1*, but at low levels according to smRNA-FISH data (Supplementary Fig. 10c,d). Among genes differentially expressed between the corpus tissue and tunica clusters, we found an *ARGONAUTE* gene (2HG0125150) related to *OsAGO14* (Supplementary Fig. 10d). *HvKN1* expression patterns in the tunica thus resemble those of *OSH1* in rice, with minimal expression in the vSAM, but higher levels in reproductive meristem tips.

In the IM, *HvKN1* is absent in founder cells (Fo) of the TSM primordium, providing an early marker for Fo cell specification (Fig. 3b,f–h). The TF encoded by *HvHD-zip*/*HvHDZIV2*/*HvOCL2* (2HG0187460) was previously reported as an IM or spike tip marker, but our smRNA-FISH data showed that it is also expressed at the tips of other meristems within the inflorescence^48^ (Fig. 3f,g). Pre-patterning of Fo cells into TSM and suppressed bract becomes apparent from Fo3 onwards. TSM founder cells lack *HvKN1*, but express *VRS4* (3HG0233930) and *HvLOG1* (5HG0502720), or upregulate *CRABS CLAW* (*HvCRC*, 4HG0396510) where the suppressed bract will form (Fig. 3b,g,h). Once the TSM visibly protrudes from the IM, *HvKN1* and *HvHD-zip* (*HvHDZIV2* or *HvOCL2*) are re-expressed within the TSM. Within the growing TSM, the expression of *HvCOM1* (5HG0479720), encoding a TCP-like TF, marks the formation of the rachilla (Fig. 3b,g,h). Using these expression profiles, we were able to isolate the TSM Fo (Fig. 3i) and identify genes specifically expressed in these cells, including an *Argonaute* gene (4HG0334490), and those encoding HD-ZIP (6HG0611880), MADS-box (*HvMADS84*, 1HG0054230), AT hook motif DNA-binding (7HG0647300) and MYB (7HG0698540) TFs (Fig. 3j and Table 11). We used smRNA-FISH data to characterize the TSM Fo in detail (Fig. 3,g,h,k), and isolated Fo, IM and P1 cells by virtual microdissection in BARVISTA. The analysis of DEGs identified candidates that were expressed in each cell population (Fig. 3l–n and Supplementary Table 12), including 27 expressed specifically in Fo cells. The latter included DEGs encoding a leucine-rich repeat protein kinase-like receptor (2HG0108610, Fig. 3m), two BREVIS RADIX-like transcriptional regulators (2HG0193530 and 5HG0535760)^49^ and two YABBY TFs (*YABBY1*/*TONGARI-BOUSHI1-related*, 2HG0184460 = *HvTOB1* and *YABBY15*/*TOB2-related*, 6HG0598850 = *HvTOB2*), both involved in node/internode specification in rice and regulated by the homeodomain TF OsH1 (ref. ^47^). *HvTOB2* was detected using both approaches, suggesting YABBYs are involved in the specification of TSM Fo cells. Among the markers for IM, we found *HvFT2* (3HG0244930), a homologue of *OsFT-L1*, which is activated in rice IM by the mobile paralogues *OsHd3a* and *OsRFT1*. *OsFT-L1* is also expressed in the undetermined primary and secondary branch meristems, but not in mature IM^50^. The function and expression profile of *HvFT2* in developing barley spikes are unclear. Another flowering regulator, the bZIP TF *HvFD7* (2HG0111000) was enriched in the IM, similar to its rice orthologue *OsbZIP62*, which physically interacts with OsFT-L1 (ref. ^51^). Primordia also express one of the two *AtSCR* homologues, *HvSCR1* (4HG0353780). In *Arabidopsis*, *AtSCR* is involved in the establishment of primordia and is expressed in FMs as early as P1 (ref. ^52^).

## Gene expression programs governing meristem and floral organ identity

**Fig. 4:**
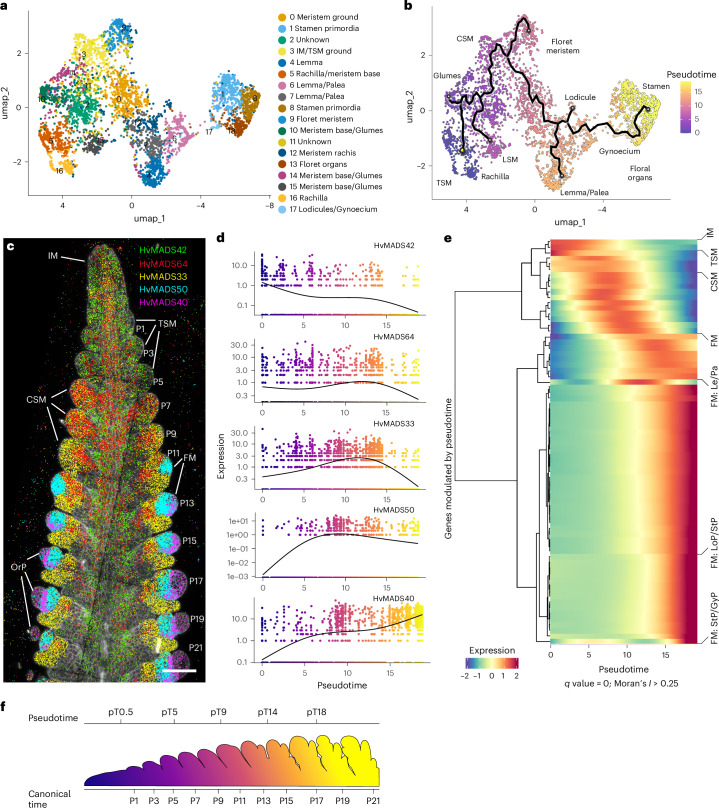
Subclustering in corpus cells postulates MADS-box transcription factor genes as markers for specific meristems and organ formation. **a**, UMAP and subclustering of corpus cells from developing spikes in barley. **b**, Pseudotime analysis in subclusters from corpus cells defines the formation of meristems and organs from inflorescence meristem to organs in the floret meristem (TSM, CSM). The yellow dot shows the starting point in pseudotime, the black line shows the developmental trajectory across the pseudotime, and endpoints for primordia formations are shown as circles. **c**, Expression profiles of selected MADS-box transcription factor genes along the developing spike. OrP, floret organ primordia. **d**, Expression profile according to pseudotime analysis for selected MADS-box transcription factors. **e**, Genes with the highest differential expression according to pseudotime analysis grouped by hierarchical clustering (Moran’s *I* ≥ 0.25 and *q* = 0), showing the major developmental transitions during spike development. **f**, Inferred equivalency of pseudotime and canonical time, as a plastochron, based on genes modulated by pseudotime. smRNA-FISH section in **c** is representative of at least 5 independent developing spikes. Scale bar, 100 µm (**c**).

**Fig. 5:**
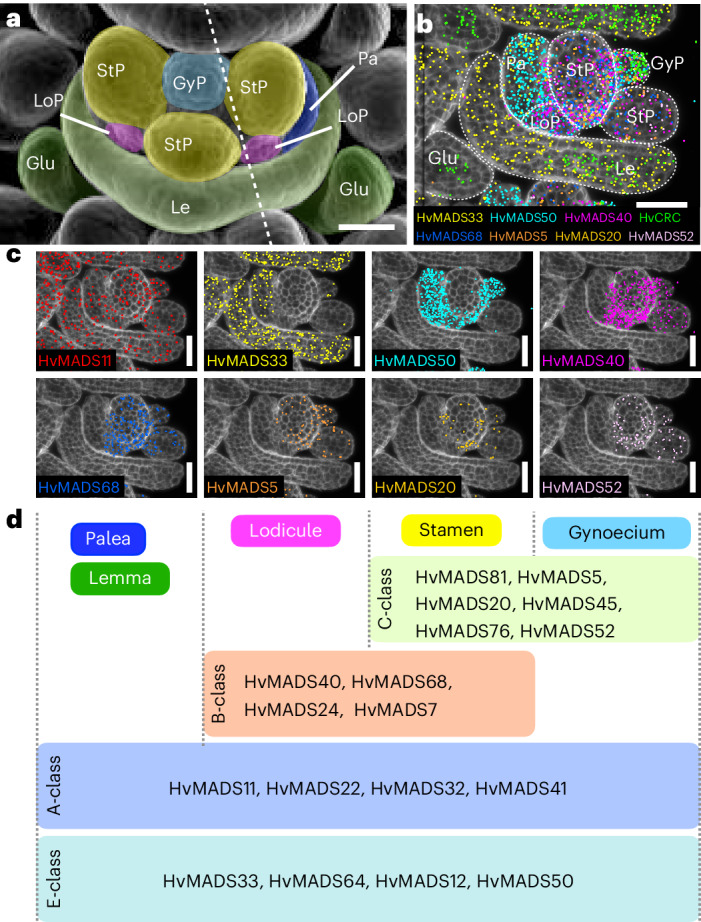
Inferred roles of *HvMADS* genes in the ABCDE model of whorl organization. **a**, SEM of one floret. **b**, Expression profiles of *HvMADS* genes in floret organs (glumes, Glu; lemma, Le; palea, Pa; lodicule primordium, LoP). **c**, Individual expression profiles of selected *HvMADS* genes. **d**, Putative role of MADS-box transcription factors inferred by pseudotime analysis in the ABCDE model of floret whorl formation. The dashed line in **a** represents the approximate orientation in the section in **b**. smRNA-FISH sections are representative of at least 2 independent developing spikes. Scale bars, 50 µm (**a**–**c**).

Subclustering in the vasculature revealed cell populations not previously detected by our general analysis (Fig. 2b), including phloem cells (Supplementary Figs. 11 and 12, and Table 13). We therefore analysed meristem-to-organ transitions in developing spikes, focusing on the highly diverse corpus cells, thereby avoiding developmental trajectories in multiple directions (for example, leading from initial cells to vasculature, tunica or corpus cells simultaneously) (Fig. 2b,c, and Supplementary Figs. 2 and 3). Within the corpus, we identified 18 new subclusters, including those annotated as TSM, CSM, FM and primordia of developing floret organs, such as glumes, lemma, palea, lodicules and developing stamens (Fig. 4a, and Supplementary Fig. 13a and Table 14). To identify genes modulated along the developmental trajectory from IM to floret organs, we applied pseudotime analysis starting from cells expressing *COM1* and *VRS4* (subcluster 16, Fig. 4a,b and Supplementary Fig. 13b,c), as bona fide markers for the establishment of SMs (Fig. 3g). The inferred pseudotime trajectory was used to track the establishment of CSM and LSM from TSM, from CSM to FM, and from FM to organ development (Fig. 4b and Supplementary Fig. 13d–s). We then searched for potential master regulators of developmental progression by focusing on key TFs whose expression commences at the branch points of the pseudotime trajectory. Among the genes detected in corpus subclusters, we found 46 MADS TF genes, 26 of which were differentially expressed between 0.24 and 74.7% of the 4,807 cells in the corpus (Fig. 4c,d and Supplementary Table 15). *HvMADS42*/*MADS34* and *HvMADS64*/*MADS5* are activated early and mostly co-expressed in the IM and TSM. *HvMADS42* expression declines following the differentiation of the CSM, and later localized to the adaxial side. *HvMADS64* and *HvMADS33* are both expressed in the CSM, but only *HvMADS33* will also mark the developing lemma and palea. *HvMADS50*/*MADS6* expression arises in the established FM and, similar to *HvMADS33*, continues in the lemma and lodicule primordia. *HvMADS40*/*MADS8* is expressed in floret organ primordia, including lodicule, stamen and gynoecium primordia (Figs. 4c,d and 5a,b). Twelve MADS TF genes were strongly modulated by pseudotime (Moran’s *I* ≥ 0.1 and *q* ≤ 3.4 × 10^−162^, Supplementary Fig. 14 and Table 16) and their expression profiles correlate with their developmental activation, thus highlighting two axes, one from the apex to the base of the spike, and another from the base to the tip in the FM developing floret organs. According to pseudotime (pT) analysis (Fig. 4f), we identify six trends in gene modulation, with gene expression peaking around major meristem transitions such as IM to TSM (pT0.5), TSM to SM (pT5), SM to FM (pT9), FM to lemma and palea primordia (pT14), and two from FM to stamen, gynoecium and lodicule primordia, one group of genes starting earlier than the second group, but both having a late peak (pT18) (Fig. 4c–f). Clustering of modulated genes by pT revealed two main clades: those expressed between IM to FM, and those involved later in floret organ organization. The first clade showed more complex gene expression patterns (IM to FM) than the second (FM to floret organ formation), whereas organ development involved more modulated genes but only two patterns in late modulation (pT >14 or >18) (Fig. 4e). We found that 20 of 26 MADS TF genes were modulated during and after the establishment of the FM, consistent with the proposed role of these TFs to define the floral whorls after floret transition (Supplementary Fig. 14). On the basis of the pT activation profiles, we can infer which other MADS TF genes are expressed in specific floral whorls (Fig. 5 and Supplementary Fig. 14). Grasses follow the ABCDE model of floral development, where organ determination is mediated by MADS TF multimers^53,54^. By analysing the detailed expression profiles of 12 MADS TF genes, we were able to assign a putative A, B, C or E class to all such genes expressed in corpus subclusters and modulated by pT, taking as a reference the proposed model for rice^55^ (Fig. 5c,d and Supplementary Fig. 14). On the basis of genes modulated during major transitions, we can correlate pT and canonical time expressed as plastochrons (Fig. 4f). Within corpus subclusters, 4,733 cells expressed at least one of the 26 HvMADS -TF genes (median = 5). By analysing the presence or absence of these HvMADS TF genes and their pT classes, we could then assign each cell to specific organs within florets (Supplementary Fig. 15), also revealing the transcriptional profiles for specific floral organs at incipient stages.

## Gene expression patterns in distinct phases of meristem development

**Fig. 6:**
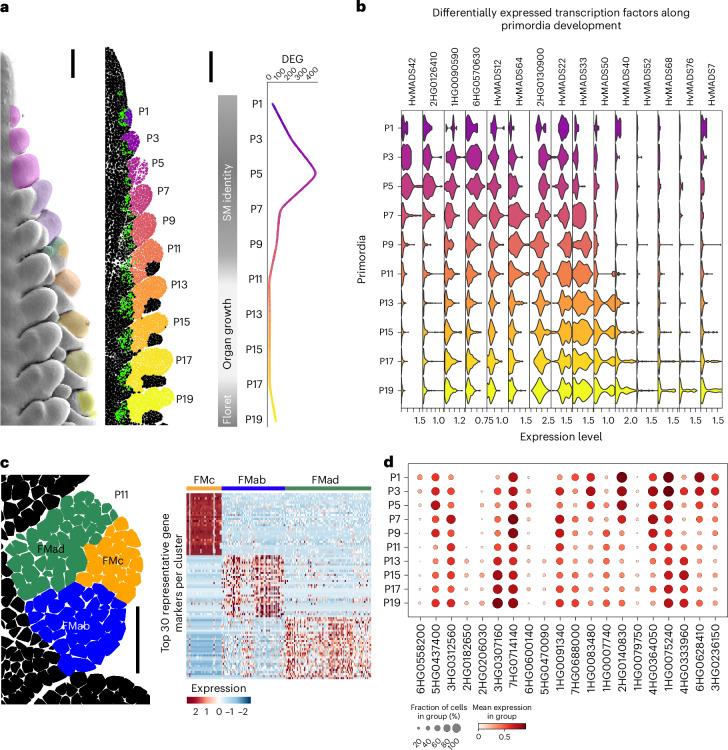
Isolation of stage- and domain-specific genetic determinants using BARVISTA*.* **a**, Apical–basal gradient of spike development: formation of TSM (P1–P3), transition (P5) to CSM (P7–P9), establishment of FM (P11–P13) and floret whorl organization (P15–P19). *VRS4* expression (green) was used to delimit primordia boundaries. P5 shows the higher diversity in imputed DEGs. **b**, Expression levels of selected DEGs encoding transcription factors across primordium development. **c**, Left: floret meristem divided into central (FMc), adaxial (FMad) and abaxial (FMab) domains. Right: heat map with the top 30 DEGs for each FM region. The heat map shows selected examples of imputed DEGs using one-sided MAST, with *P*_adj_ < 0.01 and with enrichment for cells that express genes at double or more the level of the remaining cells (pct.1/pct.2 ≥ 2). Scale bars, 100 µm (**a**); 50 µm (**c**). **d**, PERSIST identified 20 genes with specific combined expression patterns for each primordia stage.

Using the BARVISTA virtual microdissection tool, we integrated the pT data from the scRNA-seq dataset with our imputed gene expression dataset and identified genetic determinants that are both temporally and spatially associated with specific developmental stages of primordia. Barley spikelet primordia develop in a distichous arrangement, with a developmental gradient along the apical–basal axis. We selected the first 10 primordia on a single side of the sections (P1–P19, only odd numbers), and captured spatio-temporally defined DEGs throughout spikelet development from the TSM to FM and the formation of floral organs. Each spikelet was defined by excluding the subtending lemma primordium, characterized by the expression of *HvCRC*, and cells expressing *VRS4* were used to delimit spikelet boundaries (Fig. 6a). We then identified 637 marker genes by comparing their expression levels in each primordium with their expression in the total number of selected cells (Fig. 6a and Supplementary Table 17). Most DEGs were detected in the early stages P1–P9, reaching a peak of 303 DEGs in P5, which marks the transition between TSM and CSM/LSM. No DEGs were detected from P11 to P15, and the number of DEGs gradually increased from P17 (5) to P19 (150). This allowed us to subdivide spikelet development into three phases: first, SM identity is defined by transcriptional reprogramming in cells of the early spikelet primordia, which differentiate into various tissue types characteristic of the SM, becoming morphologically distinct at P11; second, rapid growth occurs during P11–P15 without significant changes in gene expression; and third, the FM undergoes differentiation, with changes in the gene expression marking the differentiation of the floret organs (GyP and StPs) (Fig. 6a). Among the DEGs, we identified 36 TF genes expressed at specific spikelet development stages (Fig. 6b and Supplementary Table 18). Corroborating our pT data, *HvMADS42* (5HG0511250) was upregulated in P1–P7, *HvMADS33* (4HG0396400) in P7–P11 after the TSM-to-CSM transition, and *HvMADS50* (6HG0604360) from P11 onwards, when the FM is established. Other DEGs in P5 included those encoding nuclear factor Y subunit B-10 (*HvNF-YB10*, 2HG0126410), two uncharacterized basic leucine zipper domain (bZIP) TFs (6HG0570630, 7HG0746810) and the ethylene-responsive TF HvERF61 (1HG0090590). *HvMADS64* (7HG0654930) was significantly upregulated in the CSM (P7). Between P11 and P17–P19, we observed organ growth, but no specific marker genes were detected until floret organs started to differentiate (P17–P19). The TF genes characterizing floret development included *HvMADS7* (1HG0065060), *HvMADS20* (3HG0243770) and *HvMADS76* (*HvMADS16*, 7HG0721170). The transcriptional and morphological similarity of primordia from P11 to P15, after the prominent transcriptional reprogramming during early spikelet development, indicated the establishment of transcriptionally defined spikelet organs at P11. To test this, we used BARVISTA to select cells from the adaxial part of spikelet P11 (FMad), which forms the rachilla, from the floret meristem (FM) and from the abaxial side (FMab) where the lemma is initiated (Fig. 6c and Supplementary Table 19). We found two YABBY TF genes as markers for FMab: *HvCRC (*4HG0396510*)*, the orthologue of *DROOPING LEAF (OsDL)* in rice, *DL2* in maize and *CRABS CLAW* in *Arabidopsis*; and *HvTOB1 (*2HG0184460*)*, the orthologue of *ZmYABBY14*/*TOB1*/*ABNORMAL FLOWER ORGANS*, all related to leaf-like organs such as the lemma in barley^56–61^. On the adaxial side (FMad), we found WUSCHEL-RELATED HOMEOBOX PROTEIN (*HvWOX3*, 1HG0010970), the orthologue of *LATERAL LEAF SYMMETRY*/*OsWOX3* in rice, *ZmWOX3a*/*ZmWOX3b* in maize, and *PRESSED FLOWER* in *Arabidopsis*, which are involved in lateral organ formation^62–64^. Other markers included *HvMONOCULM1* (7HG0720900), the GRAS-TF gene related to *ZmGRAS43* in maize, *MONOCULM1* in rice and *LATERAL SUPPRESSOR* in *Arabidopsis*, which contribute to axillary meristem formation and branching in rice and *Arabidopsis*^65–67^. In the central domain (FMc), the top candidate was *HvMADS50* (6HG0604360), related to rice *OsMADS6*/*MOSAIC FLORAL ORGANS1*, maize *BEARDED EAR-1* and *Arabidopsis* *AGL6*, which supports floral meristem establishment^68–70^. We also detected *HvLEAFY* (2HG0194240)^71^ and 59 transposon-related genes specifically expressed in the FMc.

These results show how we can use BARVISTA to subdivide spikelet development into different transcriptionally distinct phases and identify relevant TFs marking the differentiation of specific spikelet organs even before they become morphologically distinct. We then used PERSIST to identify gene expression combinations suitable as barcodes for stages P1–P19 (ref. ^46^) (Fig. 6d and Supplementary Table 20). The barcode can be used to rapidly assign a cell of unknown origin to the correct primordium, on the basis of the similarity of gene expression to the identified barcode genes. Such a barcoding system will determine single-cell identities more precisely in future experiments, estimating a cell’s position within a tissue even in the absence of spatial experimental data.

## Characterizing mutant phenotypes at the single-cell level via gene expression

**Fig. 7:**
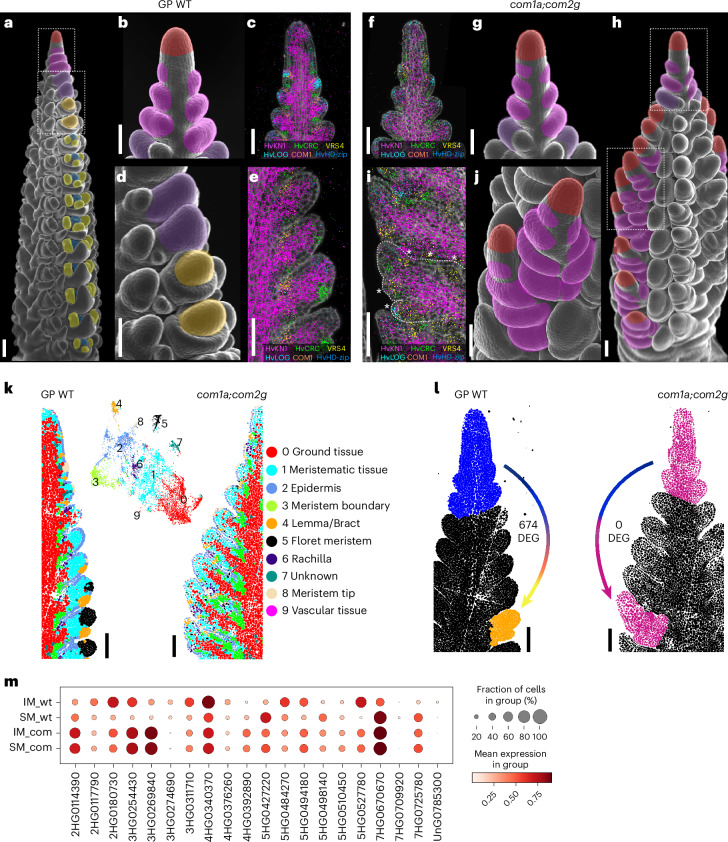
Imputed differential gene expression for molecular phenotyping at the single-cell level. **a**, SEM of barley spike indicating the IM (red), TSM (pink), CSM (orange), stamen primordia (yellow) and gynoecium primordia (blue). **b**, Close-up of one developing spike highlighting TSM formation, equivalent to the top dashed box in **a**. **c**, Gene expression patterns associated with the establishment of TSM. **d**, Close-up from **a** showing the CSM and its transition to FM. **e**, Marker gene expression profiles during the transition from TSM to FM. **f**, Marker genes expressed in the top part of a *com1a;com2g* developing spike. **g**, SEM of an equivalent region as shown in **f**. **h**, Developing spike of *com1a;com2g*; initiation of TSM (top dotted box) and further IM-like development (bottom dotted box). **i**, Expression profile of marker genes in an IM-like branching structure, with asterisk indicating the formation of ectopic SMs. **j**, SEM of branched structure equivalent to the bottom dotted box in **h**. **k**, Spatial clusters of smRNA-FISH data from WT and *com1a;com2g* after integration. The WT SMs acquired FM and floret expression profiles (cluster 5 FM, black cells). In *com1a;com2g*, cells with FM profile (black, cluster 5) are underrepresented and branches maintained tip-like expression profiles. **l**, Imputed DEGs between the upper part of the spike, including the IM and the first four primordia (P1–P4, blue), and P13 (FM, orange) in WT, and its counterpart in *com1a;com2g*., colour gradient in the arrows suggest the progression along the pT. **m**, Gene expression barcodes for SM and IM of WT and *com1a;com2*g (as selected in **l**). The barcodes show expression levels of 20 genes that distinguish between the tissues. Genes were selected using PERSIST, solely on the basis of their expression patterns. smRNA-FISH sections are representative of at least 2 independent developing spikes. SEM WT images are representative of their developmental stage, *com1a;com2g* images are representative of 10 spikes analysed. Scale bars, 100 µm.

Spikelet identity is determined by the TFs COM1 and COM2, which act in partially independent pathways. Plants carrying mutations in both *COM1* and *COM2* develop branched inflorescences, which was interpreted to be a reversion of CSMs into indeterminate IM-like structures that generate multiple spikelets on their flanks^9,72^ (Fig. 7a,b,d,g,h,j). Using expression markers for spikelet formation (*VRS4*, *HvCRC* and *HvLOG1*), we compared gene expression profiles in the apical and lateral meristems between WT and *com1a;com2g* inflorescences. Early expression profiles appeared identical (Fig. 7c,f), but when WT CSMs generated different spikelet organs, *com1a;com2g* CSMs grew indeterminately, and the onset of *VRS4*, *HvCRC* and *HvLOG1* expression in the *com1a;com2g* background was reduced and delayed (Fig. 7e,i; white asterisks), resembling expression patterns of WT inflorescence tips. We integrated smRNA-FISH data from WT and *com1a;com2g* spikes and identified cell-type-specific clusters on the basis of similar gene expression profiles, which were mapped onto the corresponding spike section. At the gene expression level, WT SMs gradually acquired FM and floret profiles, whereas cells in *com1a;com2g* branches maintained tip-like profiles (Fig. 7k).

We identified transcriptional signatures underlying spikelet determination by imputing gene expression differences between the inflorescence tips, spikelets and the *com1a;com2g* IM-like branches. The IM-like branch bears four axillary SMs, so we selected the inflorescence tip including the first four spikelet primordia (Fig. 7l). In WT plants, we identified 674 DEGs between the inflorescence tip and determinate spikelet, whereas *com1a;com2g* branches showed no DEGs in comparison to the inflorescence tip (Supplementary Table 21). This demonstrates how specific transcriptional signatures can be used to describe phenotypic profiles, independent of morphological comparisons. We then used PERSIST to generate gene expression barcodes for WT and mutant inflorescence tips and AMs. WT inflorescence tip barcodes were clearly distinct from those of the determinate spikelet, whereas those of the *com1a;com2g* inflorescence tip and branches were indistinguishable. Interestingly, expression barcodes for the mutant inflorescence tip and branch shared commonalities with both WT inflorescence tip and spikelet, indicating that both *com1a;com2g* inflorescence tip and branches could represent an intermediate state at gene expression levels. Comparison of MADS-TF gene expression revealed that *COM1* and *COM2* promote *HvMADS33* and *HvMADS64* expression from P7 onwards, and *HvMADS20* and *HvMADS50* expression in FMc from P11 onwards (Supplementary Fig. 16).

Complete gene expression profiling and tissue/stage-specific expression barcodes thus facilitate the characterization of mutant phenotypes and underlying changes in gene expression, and the mapping of cell and tissue expression profiles in complex genetic backgrounds.

## Extending spatial gene expression datasets via gene expression imputation

A cell´s location in plant tissues is generally fixed, but cells can be passively displaced during growth while keeping contact with their neighbours, establishing local clones. Positional information is imparted by the perception of juxtacrine or symplasmic signals from direct neighbours and/or by exposure to specific concentrations of morphogens or abiotic factors such as light. Such spatial inputs modulate the same gene expression network operating in different cells and can generate different outputs depending on a cell’s position. However, spatial transcriptome methods cannot yet accommodate the full complexity of transcripts in a cell. For probe-based methods, this reflects the molecular crowding of bulky probes, which are needed for sensitivity or specificity of target RNA detection. Methods based on cDNA sequencing are limited by the efficacy of RNA capture from tissues. Both methods generally detect transcripts from fewer than 5,000 genes, with limited information on expression dynamics. Alternatively, scRNA-seq reveals the expression dynamics of many thousand genes, without spatial information. Our approach combines the benefits of both methods to address the problem of space in gene expression studies^32^. We obtained cell-resolved, quantitative data on gene expression, covering a maximum number of genes, by combining scRNA-seq and cell-resolved transcript detection on tissue sections. Using a small set of probes as anchors to reliably detect transcripts at many different expression levels, we matched cells from tissue sections with those from the scRNA-seq dataset, using CS followed by calculation of a weighted average from the most similar cells for each gene. We then used gene expression information from the scRNA-seq dataset to impute the missing expression values of almost all barley genes for all cells in the tissue sections. Validation tests showed that the imputed gene expression values were highly reliable, after exclusion of protoplasting-induced genes. We focused on key imputation parameters to identify our method’s limitations, and found that the 81 anchor genes from the smRNA-FISH experiment were able to identify similar cells between the datasets; however, the selection of anchor genes could be optimized using the deep learning tool PERSIST for gene selection. For a dataset as complex as the barley inflorescence, scRNA-seq data from 10,000 cells and 20 anchor genes were sufficient for gene expression imputation. Gene combinations can be used to differentiate between cell populations and to establish simplified gene expression barcodes for specific cell identities or states in a dataset, characterizing complex changes in gene expression in mutants or during developmental progression. Future applications could integrate additional data types, such as proteomic or metabolomic data. Within the given experimental limitations, our imputation approach enables gene expression analysis by virtual microdissection of individual cells for the discovery of cell- or location-specific characteristics, or to determine functional domains within meristems that underlie specific traits. Recent studies combined co-expression and orthology^73^ to identify tissue-specific genes and markers across plant species^22,27,29^. Our dataset can serve to implement cross-species gene imputation to generate a spatial pan-grass transcriptome.

## Dynamic gene expression underlying the formation and specification of primordia

*KN1* and its orthologues promote meristem fate in many cereal species, and we found that the downregulation of *HvKN1* combined with *HvHD-zip* expression is a hallmark of primordia initiation in the vSAM and IM. Leaf founder cells were predominantly marked by expression of *HvCRC*, which was also expressed in spikelet founder cells, but polarized to the abaxial side marking cells that form the suppressed bract. The adaxial side expresses *HvLOG1* and the TFs *VRS4* and *COM1*, which promote spikelet determinacy.

**Fig. 8:**
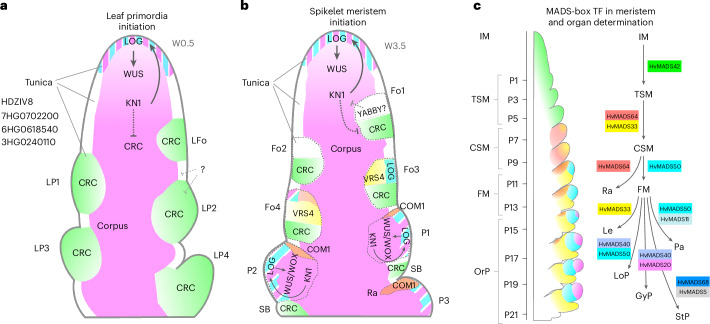
Model for primordia initiation and meristem organization in vegetative SAM and developing inflorescences. **a**, Model for leaf primordia specification and initiation in vegetative SAM. HvKN1 (magenta) is expressed in the corpus and the tip of the SAM; HvKN1 positively regulates LOG (magenta and cyan stripe pattern), which promotes cytokinin biosynthesis. Cytokinin upregulates HvWUS/HvWOX genes to organize meristem function. Leaf primordia are specified in founder cells (LFo) depleted for HvKN1, with HvCRC (green) as the earliest sign of leaf primordia determination (LP). **b**, Inflorescences initiate triple spikelet meristems (TSM). Founder cells (Fo1–Fo2) lack HvKN1 expression; HvCRC (green) in the basal domain marks Fo cells of the suppressed bract (SB), YABBY TFs become expressed in the apical domain. At Fo3–Fo4, VRS4 (yellow) and LOG (cyan) are expressed in the apical domain, possibly specifying TSM identity. From P1 onwards, HvKN1 expression is restored and extends into the tunica. LOG expression fosters cytokinin accumulation, which upregulates HvWUS/HvWOX genes to organize and pattern the newly formed TSM. Expression of COM genes (orange) then determines the rachilla meristem (Ra). **c**, MADS TFs are major regulators in meristem and floret organ determination. HvMADS42 (green) is expressed during the transition from IM to TSM. HvMADS64 and HvMADS33 (red and yellow, respectively) are expressed in CSM, then HvMADS64 specifically in Ra. HvMADS50 (cyan) is specific for the central zone of FM, HvMADS33 for the lemma primordia (Le), the first floral organ formed. Combinations of HvMADS TF genes mark the specification of other floral organs (OrP). HvMADS50 (cyan) and HvMADS11 are expressed in the palea (Pa); HvMADS50 and HvMADS40 in the lodicule primordium (LoP); HvMADS40 and HvMADS20 in the gynoecium primordia (GyP) and HvMADS68 and HvMADS5 in stamen primordia (StP).

*KN1* mRNA in meristems was restricted to corpus tissue and only sporadically found in the tunica layers of maize or rice. We found that *HvKN1* is expressed in the corpus of spikes and, at a lower level, of vSAMs. *HvKN1*-positive cells from the corpus of vSAM, and *HvKN1*-negative tunica cells from spikes showed positive correlation in gene expression, indicating shared developmental paths (Supplementary Fig. 10e,f). *HvKN1*-positive tunica cells are located at the tips of IMs and SMs, whereas tunica cells in leaf primordia tips of the vSAM lacked *HvKN1* expression. Expressions of *HvKN1* and *HvLOG1* correlate and might generate a cytokinin source in meristem tip tunica cells (but not those in the tips of leaf primordia). Hypothetically, such a local cytokinin peak could establish the *WUS/WOX* expression domains in the IM and newly formed SMs^74^ to provide a feedback signal that maintains *HvKN1* expression, thereby acting as a local organizer that promotes meristem maintenance from the outermost cell layer (Fig. 8a,b).

Virtual microdissection using BARVISTA identified specific marker genes for the IM, TSM founder cells and TSM primordia (Fig. 3f–h). These included genes encoding the flowering regulators HvFT2 and HvFD7 for IM, the TFs HvTOB1 and HvTOB2 for Fo cells, and HvSCR1 for P1 or TSM. In maize, stem cells in the vSAM and IM, marked by *ZmWUS1* and *ZmCLE7* expression, are often difficult to recover from protoplast preparations^22^. Our scRNA-seq dataset included a significant number of cells expressing *HvWUS1* and *HvCLV3*. Furthermore, maize *PYRUVATE ORTHOPHOSPHATE DIKINASE* (*ZmPPDK*) is highly co-expressed with *ZmCLE7* in the IM^22^, and we found *HvPPDK* (1HG0062940) transcripts enriched in the barley IM, indicating that expression imputation can digitally reconstruct the IM and facilitate marker gene identification from limited tissues. Finding marker genes specific for the IM is challenging because many genes relevant for IM development are also expressed in SMs, including *HvKN1*, *HvLOG1* and *HvHD-zip*/*HvHDZIV2*/*HvOCL2* (Fig. 3g), or *HvMADS42*/*MADS34* and *HvMADS64*/*MADS5* (Supplementary Fig. 14). Alternatively, the expression dynamics of several genes can be combined into highly specific expression profiles using a barcoding strategy to identify specific cell types or tissues.

## Subclustering and pseudotime analysis predict meristem transitions and the path to floret organ establishment

Barley spikes at W3.5 comprise all major stages and transitions in the developmental path towards floret formation. We used *COM1* expression in the TSM as a starting point for pseudotime analysis. Double mutants of *COM1* and *COM2* develop branched spikes, suggesting a defect in early stages of CSM determination^9^. *VRS4* is already expressed in TSM Fo cells (Fig. 3g) and follows a pseudotime expression profile similar to that of *COM1* (Supplementary Fig. 13b,c). Following the developmental trajectory by pseudotime analysis, we identified genes marking the major transitions from IM to floret organs in the inner whorl. Genes modulated during the transitions from IM to FM (up to pT9) showed a higher diversity in patterns of gene modulation. Later in development, from FM to the formation of floret organs, patterns were less diverse, but more genes were modulated (Fig. 4e and Supplementary Table 16). We then reconciled pseudotime and canonical time using the plastochron and developmental time based on meristem or organ formation (Fig. 4f). MADS TFs are master regulators of floret organ development, and by using their pseudotime modulation, we assigned them to the ABCE model of floret development (Fig. 5). Virtual microdissection with BARVISTA corroborated the pseudotime analysis data and identified potential regulators of key developmental transitions. Many DEGs were detected at stage P5 when the TSM separates into a determinate CSM and LSMs, including *HvNF-YB10*, *HvERF61*, *HvMADS33* and *HvMADS42*. *Arabidopsis* NF-Y mediates epigenetic control of the floral transition^75^, and an *HvERF61* orthologue regulates key floral constituents in *Osmanthus fragrans*^76^. Mutation of *HvMADS33*/*MADS1* generated branched spikes at high temperatures^11^, and the mutation of *Setaria italica SiMADS34* (*HvMADS42)* generated panicle-like inflorescences with long primary branches^77^. Rice *OsMADS34* cooperates with *OsMADS5* to promote meristem determinacy, and *osmads5;osmads34* double mutants generate multiple branches^78^. *HvMADS64* (*OsMADS5)* was upregulated in the WT CSM (P7), while the branched inflorescences of *com1;com2* mutants did not express *HvMADS20*, *HvMADS33*, *HvMADS50* and *HvMADS64*. We conclude that a key function of TFs expressed from P5 onwards is specification of CSM identity and branch suppression. *HvMADS7* (*AtPISTILLATA*), *HvMADS20* (*OsMADS3*) and *HvMADS76*, which regulate petal, carpel and stamen development in *Arabidopsis*, rice and barley^79–81^, are probably stage-specific master regulators of floret organ development (Fig. 8c).

We have showcased how the computational integration of scRNA-seq and spatial transcriptomics data significantly advances our understanding of plant meristem development and organ formation, allowing us to identify and study the underlying gene regulatory network. The gene expression imputation approach that we developed closes a gap in our understanding of developmental processes by allowing us to deduce quantitative expression data for most genes with cellular resolution. High costs associated with spatial and single-cell transcriptomics can be reduced using imputation approaches, with the aim to generate multidimensional atlases for developmental and physiological studies in plants. BARVISTA allows the intuitive navigation of these data to identify gene expression patterns of specific genes, individual cells or cell groups with high spatial resolution. Combined with functional dissection of promoter elements, we should soon be able to redesign cereal inflorescence architectures and adapt crop plants to new challenging environments.

BARVISTA currently offers only a snapshot of barley development, with expression data for specific developmental time points limited to two dimensions. We will now aim to reach beyond the intrinsic limitations of two-dimensional spatial analysis, towards three- and four-dimensional (time) reconstruction of barley development. Furthermore, cell shape, cell size and cell neighbourhoods will be considered to enable a deep understanding of cell identity and function within different spatial contexts^82^.

## Methods

### Plant growth conditions

WT tissue was collected from *Hordeum vulgare* cv. Golden Promise plants grown in Minitray soil (Einheitserde, Einheitserde Werkverband) in 96-well trays with 4 g l^−1^ Osmocote Exact Hi.End 3-4 M 4th generation (ICL Group) under long day conditions (16-h photoperiod, 20/16 °C day/night temperature). Before seed sowing, grains were pre-germinated for 4 days at 4 °C in Petri dishes lined with wet paper. Golden Promise takes ~30 days after emergence to reach W3.5 under these conditions. For the scRNA-seq work, 25 biological replicates per sample were collected and pooled, and the overall experiment was carried out three times. The *com1a;com2g* mutants were grown under the same conditions and collected when WT (Bowman) reached W3.5. We dissected ~80 plants for vSAM library preparation 11 days after sowing in soil. The tissue was collected for protoplast isolation before verification at the W0 stage.

### Protoplast isolation

Protoplasts were generated from developing inflorescences (W3.5, when the stamen primordia emerged). The tissue was directly collected in 6-well plates containing 3 ml washing solution I (WSI; 0.4 M mannitol, 20 mM MES, 20 mM KCl, 10 mM CaCl_2_, 0.1% BSA, pH 5.7). For cell wall digestion, the tissue was chopped in WSI using a scalpel and immediately digested for 140 min in 4 ml enzyme solution (0.4 M mannitol, 1.2% Cellulase R-10, 1.2% Cellulase RS, 0.36% Pectolyase Y-23, 0.4% Macerozyme R-10 (all from Duchefa), 20 mM MES, 20 mM KCl, pH 5.7, 10 mM CaCl_2_, 0.1% BSA, 0.06% β‑mercaptoethanol). Protoplasts were then passed through a 40-µm cell strainer (Scienceware Flowmi) into microfuge tubes for centrifugation (250 *g*, 2.5 min, 4 °C). The pellets were gently washed once with WSI and twice with WSII (0.6 M mannitol, 2 mM MES, 0.1% BSA, pH 5.7) with centrifugation under the same conditions described above between each wash. The protoplasts were passed through the 40-μm cell strainer and stained with calcein AM (10 μM) and DRAQ7 (1.5 μM) for 5 min at room temperature. An aliquot was loaded into a haemocytometer to score cell viability using a BD Rhapsody scanner. Only samples exceeding 70% viability were loaded into the BD Rhapsody Express system for partitioning and mRNA capture.

### scRNA-seq

We loaded ~20,000 cells per replicate. Single-cell libraries were prepared using the mRNA Whole Transcriptome Analysis (WTA) kit (BD-Biosciences) and libraries were sequenced on the Illumina NextSeq2000 platform, aiming for 50,000 reads per cell. Raw scRNA-seq data were analysed using the BD WTA Rhapsody analysis pipeline. Gene reads were aligned to the *H. vulgare* cv. Morex V3 reference genome (10.5447/ipk/2021/3).

### Subtraction of DEGs induced during protoplast isolation

Total RNA was extracted from three replicates of fresh protoplasts and intact inflorescences using the Direct-zol RNA Miniprep Plus kit (Zymo Research), followed by bulk RNA sequencing using the Illumina NextSeq platform (Biomarker Technologies). Reads were aligned to the *H. vulgare* cv. Morex V3 reference genome and converted to transcripts per million (TPM) by adapting a custom Python script (https://github.com/cvanges/spike_development). DEGs were defined using a count-based Fisher’s exact test in the R package EdgeR v.3.32.1. The false discovery rate (FDR) was adjusted using the Benjamini–Hochberg procedure. Genes were considered differentially expressed when exceeding the threshold logFC ≥ 3 or ≤–3 and an FDR < 1 × 10^−5^. These DEGs (3,684 genes) were removed from the scRNA-seq expression matrix before identifying the most variable features for cluster analysis.

### UMAP and replicate integration

Replicates were merged using the Seurat package (v.4.0)^43^. Low-quality cells were removed with a threshold of 800 < genes < 9,000, and cells were log normalized. Protoplasting-affected genes were removed from the merged object, and the 7,500 most variable features were identified using the vst function in Seurat. Next, FindIntegrationAnchors identified anchors to integrate the three replicate datasets using 30 dimensions. The integrated expression matrix was retrieved using IntegrateData. Dimensionality was reduced by scaling the expression matrix using ScaleData, and then by principal component analysis (PCA). The top 50 components were selected for further analysis. Cells were clustered using a *k*-nearest neighbours and SNN graph method. FindNeighbors and FindClusters were applied with a resolution of 1.3. Dimensionality reduction was then achieved using the UMAP algorithm with the top 35 principal components and a minimal distance of 0.01.

### Cluster-specific gene marker identification

Genes enriched in each cluster were identified using the FindAllmarkers function in Seurat with thresholds of: log.FC = 0.25, pct1 > 0.25, pct2 < 0.3, *P*_adj_ < 0.001. Differential gene expression was calculated using the function FindAllMarkers and MAST^83^.

### Multiplex smRNA-FISH

Developing inflorescences were fixed in phosphate buffered saline containing 4% paraformaldehyde and 0.03% Triton X-100 (Sigma) before dehydration through an ethanol series and progressive embedding under vacuum in Paraplast X-tra (Leica). We prepared 10-μm sections on glass slides (Resolve Biosciences), which were deparaffinized, gradually rehydrated and permeabilized with proteinase K (Thermo Fisher). After refixation, acetylation and dehydration through an ethanol series, the slides were mounted with SlowFade-Gold Antifade reagent (Invitrogen). Probe hybridization and imaging were carried out at Resolve Biosciences using in-house protocols. The target genes are listed in Supplementary Table 5.

### Pseudotime analysis

The Seurat object with the integrated cells was used for pseudotime trajectory analysis with monocle3 (v.1.3.7)^84–86^ in RStudio. Only clusters from corpus cells were analysed in detail. These were first subclustered with Seurat using 50 components, a resolution of 2 and a dimensionality of 35. *COMPOSITUM1* (5HG0479720) was selected as the starting point for developmental time because COM1 is specifically expressed at the branching point of developmental trajectories towards tunica, corpus or vascular fates. The subclusters were ordered according to the learnt trajectory, and pseudotime-modulated genes were calculated using graph_test with threshold values of Moran’s *I* > 0.1 and *q* = <0.01. Selected examples (q_value == 0) were visualized using ComplexHeatmap (v.2.18.0)^87^.

### Cell segmentation

To calculate the gene expression matrix for the MC data, the DAPI image was segmented using a workflow provided by Resolve Biosciences (https://my.resolvebiosciences.com/help/segmentation). Briefly, the pipeline first runs MindaGap (https://github.com/ViriatoII/MindaGap) with default settings to improve the borders of the image tiles, then Cellpose^88^ with the parameters: model=cyto, diameter=50.0 and flow-thresh=0.5. Genes with ≥70% transcripts inside DAPI segments were considered nucleus specific, and those with ≤30% transcripts inside DAPI segments were considered cytoplasm specific. Finally, Baysor^89^ was run using the DAPI roi files as prior with the parameters: –no-ncv-estimation–force-2d–n-clusters=1–prior-segmentation-confidence 0.9 -m 3, as well as the previously determined list of nucleus/cytoplasm-specific genes for –nuclei-genes and –cyto-genes. The resulting MC count matrix was used for subsequent analysis.

### Integrated clustering

Seurat v.4.3.0 was fed with count matrices from the MC and scRNA-seq as inputs. Data quality was based on the number of cells per sample, the number of Unique Molecular Identifiers (UMIs) per cell, the number of genes per cell and the complexity of all four datasets. Subsequently, all genes not recorded in the MC dataset, as well as those affected by protoplasting, were removed from all count matrices, resulting in a total of 81 anchor genes for the IM, 76 for the vSAM and 83 for the *com1;com2* mutant. The datasets were log normalized on the basis of the natural logarithm with a scale factor of 1 using NormalizeData. All four Seurat objects were merged into one using Seurat’s merge function and then split into a list object with SplitObject, with one list element containing one sample. A second normalization step was applied across all four datasets for further harmonization. All available genes were selected for subsequent integration using FindVariableFeatures and SelectIntegrationAnchors, with the identified integration features as anchor features and the MC dataset as a reference to integrate the other three datasets. For the clustering of the vSAM, we tested different parameter settings in SelectIntegrationAnchors (Supplementary Table 22). We varied k.anchor between 3 and 10, k.filter between 25 and 400, and k.score between 10 and 50. For the final clustering of the vSAM, we selected k.anchor = 4, k.filter = 25 and k.score = 30, whereas default parameters were used for the IM. The datasets were integrated using IntegrateData, scaled using ScaleData, and PCA was performed using RunPCA with VariableFeatures as PCA features and 50 dimensions. Neighbouring cells were calculated using FindNeighbors on the PCA-reduced dataset with the first 35 dimensions, and clusters were determined using FindClusters with varying resolutions between 0.1 and 2.0 (step = 0.1). The *com1;com2* mutant was clustered with a resolution of 0.5. The clusters were visualized on the basis of Seurat’s RunUMAP results, using the PCA reduction method and 35 UMAP dimensions. The differential gene expression of each cluster was compared to all other clusters using FoldChange in Seurat v.5.0.3 for each resolution (a different Seurat version was used due to a known error in the fold-change calculation of Seurat v.4.3.0). All genes from the reference genome were annotated using Mercator (v.4.6)^90^. Clusters were identified using known marker genes as well as the Mercator annotations.

### Imputation of genome-wide gene expression

For all genes in the scRNA-seq dataset (reference data) but not measured in the MC experiment (test data), gene expression was imputed in a two-step procedure, loosely as previously described^91^. All raw counts were normalized to counts per million (CPM) without additional integration. The first step of imputation (cell similarity determination) required the similarity between each MC cell and scRNA-seq cells (SCs) to be determined. We calculated the CS between each MC cell and all segmented cells within the same cluster on the basis of the 81 previously determined anchor genes, and the result was shifted by 1 into the positive range. For the vSAM, the CS to all segmented cells within all clusters was calculated instead due to the lower number of overall segmented cells. During the second imputation step, the expression of each MC cell was imputed by taking the weighted mean gene expression in the *n* = 25 most similar segmented cells using the shifted CSs from the calculated similarity matrix as weights (equations (1) and (2)). For the vSAM, the number of top *n* cells to keep was varied between 1 and 25 during an additional parameter optimization step, ultimately using *n* = 4 for the final imputation.

1 $$\it {\mathrm{weight}}_{i}={\mathrm{CS}}_{i}$$

2 $$\it \it \it \it \it \mathrm{expression}(\mathrm{gene})=\frac{{\sum }_{i=0}^{n}\mathrm{neighbouring}\,{\mathrm{Expression}}_{i} {\mathrm{weight}}_{i}}{{\sum }_{i=0}^{n}{\mathrm{weight}}_{i}}$$

### Imputation quality assessment

To evaluate the accuracy of the imputation method, the imputed gene expression values of the MC data were compared to the CPM-normalized smRNA-FISH gene expression values. The comparison of imputed and experimentally measured values was applied for the anchor genes (81 for the IM, 76 for the vSAM, 83 for the *com1;com2* mutant) using CS as the similarity measure (equation (3)). Gene expression patterns were compared on a cell-by-cell basis. For genes not measured in the MC data, the scRNA-seq data were used instead.

3 $$\mathrm{cosine}\,\mathrm{similarity}({\bf{A}},\,{\bf{B}})=\frac{{\bf{A}}{\rm{\cdot }}{\bf{B}}}{||{\bf{A}}|| ||{\bf{B}}\mathrm{||}}$$

where **A** is the gene expression vector of the experimentally measured values, and **B** is the gene expression vector of the computationally imputed values.

To further evaluate the accuracy of the method, the scRNA-seq data from the inflorescence was split into test and reference datasets using the third scRNA-seq batch containing 5,910 cells as test cells to impute gene expression values for, and the cells of the first and second batches to impute the gene expression from. The first test set contained the same 81 genes from the MC, and the second test set contained all 48,904 genes from the scRNA-seq experiment. For both test sets, the cell similarity determination step of the imputation was performed using the 81 anchor genes. During validation of the imputation for the first test set, we used the 81 anchor genes (scSub IM 81 genes), and for the second test set we used all 48,904 genes (scSub IM 48,904 genes).

### Imputation quality simulations

Gene expression imputation quality was simulated on different subsets of the scRNA-seq dataset from inflorescences to investigate the impact of different settings on the imputation quality. The full dataset consists of ~16,500 cells and 48,904 genes. Five different simulations were performed, each with five repetitions per parameter setting. Test datasets were generated containing 1,000 cells and the same genes as the reference datasets. Gene expression was validated using all 48,904 genes in the cell-by-cell comparison of imputed to experimentally measured gene expression values.

Simulation 1: Impact of reference cell number. Subsets with 100–15,000 cells were tested. Gene expression was imputed using 81 anchors.

Simulation 2: Impact of gene number. Subsets with 5,000 cells and 20–40,000 randomly selected genes were tested. Gene expression was imputed using the genes contained in the reference dataset during the cell similarity determination.

Simulation 3: Impact of the MC anchor genes on imputation accuracy, compared to the random gene selection from simulation 2. Reference subsets were generated in the same manner as for simulation 2 for combinations of 20–80 genes.

Simulation 4: Impact of gene selection method on imputation quality. Sets of 20–100 genes were identified using PERSIST^46^(https://github.com/iancovert/persist; initial release) and reference subsets generated as described above. Default parameters from the provided scripts were used, with modification of the setting flavour = ‘seurat’ for the determination of highly variable genes. During unsupervised gene selection, the setting lam_init = 0.64 was applied.

Simulation 5: Impact of gene variability on imputation quality. Reference subsets were generated containing 5,000 cells and the top 250 most or least variable genes as determined using Seurat’s FindVariableFeatures command.

### Generation of primordia barcodes

Gene expression barcodes for different sections of the IM from WT and *com1;com2* mutant plants were generated using PERSIST. One barcode was generated for the sections P1–P19 and another for the meristem tip and floret meristem of the WT and *com1;com2* mutant. During file preprocessing, highly variable genes were detected using the flavour = ‘seurat’ setting. The data were split into test and training sets to run the supervised training with default settings plus lam_init = 0.64. After training, 20 genes were selected for each barcode using default settings.

### Data pre-processing

Multiple input files were prepared for visualization based on Seurat clustering results, containing cell IDs, UMAP coordinates, cluster IDs and cluster names for all calculated resolutions. A data table was prepared containing log_2_FC and *P* values, and annotation information for each gene from *S*eurat’s FindAllMarkers for each cluster and cluster resolution, including the top-level Mercator bin in the ‘classification’ column and the leaf-level Mercator bin in the ‘annotation’ column. The count matrices were CPM normalized and rounded to zero decimal points, compressed using gzip with six compression levels and saved separately in two matrices for the MC and SC data.

Polygon coordinates were extracted in ImageJ/Fiji v.2.9.0/1.53t (Java 1.8.0_322; https://imagej.net/ij/; accessed 13 March 2024) by creating a 16-bit mask of the calcofluor image using the roi files from the cell segmentation. Briefly, the developed macro creates a new image file the same size as the original image with pixel values of zero, creating a black background. For each roi file containing one cell from the segmentation, the pixel values were changed to the cell ID, creating the 16-bit mask file. To extract polygon corner coordinates, we developed a Python script using the pillow library v.9.4.0 (https://python-pillow.org/; accessed 13 March 2024), scipy v.1.10.1 (https://scipy.org/; accessed 13 March 2024) and scikit-image v.0.19.3 (https://scikit-image.org/; accessed 13 March 2024), which extracts pixel coordinates for each cell and calculates their convex hull. Polygon corner coordinates and the pixel value as cell ID were saved in JSON format to create a suitable javascript input.

### Web application BARVISTA

For data visualization, we developed a web application in JavaScript (https://www.plabipd.de/projects/hannah_demo/Barvista.html) with D3.jsv7.8.4 (https://d3js.org/; accessed 26 October 2023) as the main framework. The package dom-to-image v.2.6.0 (https://github.com/tsayen/dom-to-image/tree/master; accessed 13 March 2023) was used for image downloading. The gzipped expression matrices were streamed from the server to the client, making use of DecompressionStream from the Compression Streams API, ReadableStream from the Streams API and TextDecoder from the Encoding API. The web application visualizes the integrated barley data and has two main plots, one displaying the cell clusters (UMAP plot) and the other displaying the cells within tissue sections. Clusters are colour coded and identified in both plots. At the bottom of plots, a data table displays information about a selected cluster, including the genes, their average log_2_FC values, the percentage of cells in the selected cluster that express each gene (pct.1), the percentage of cells in all other clusters that express each gene (pct.2) and their Mercator annotation. The table is searchable for genes or annotations of interest. The default resolution of the cluster data is 1.3, but a resolution selector allows visualization at resolutions of 0.1–2.0 to show clustering with a higher or lower number of clusters. Gene expression information can be visualized in both plots for two genes in parallel. Cells in the tissue plot can be selected with the selection tool and gene expression can be downloaded as a subset for further analysis of specific regions of interest.

### Imputed differential gene expression analysis

Subsets of cells in the tissue sections were selected using the selection cell tool in BARVISTA, aiming to capture IMs (IM-01 and IM-02), founder cells (Fo-01 and Fo-02) and the first properly organized TSM primordia cells (P1-01 and P1-02). The matrices containing the imputed counts were used to create a Seurat object and were then log normalized. Cells from each category were considered as a cluster, and the imputed DEGs were calculated using the FindAllmarkers function in Seurat and a MAST statistical method, with thresholds log.FC > 0.1, pct.1 > 0.25, pct.2 < 0.5, *P*_adj_ < 0.05 and a pct.1/pct.2 ratio >1.2.

### Reporting summary

Further information on research design is available in the Nature Portfolio Reporting Summary linked to this article.

## Supplementary information

Supplementary Information Supplementary Figs. 1–16 and Data.

Reporting Summary

Supplementary Tables Supplementary Tables 1–22.

## Data Availability

Metadata associated with this investigation can be accessed at 10.60534/zfdth-2g147 (ref. ^92^). BARVISTA is accessible at https://www.plabipd.de/projects/hannah_demo/Barvista.html.

## Code availability

The code is available upon request to the authors.
