# Supplementary material for: Imputation integrates single-cell and spatial gene expression data to resolve transcriptional networks in barley shoot meristem development
Source: Nat Plants. 2026 Jan 7;12(1):107–24. doi: 10.1038/s41477-025-02176-6 (PMC12830361; doi:10.1038/s41477-025-02176-6)
Supplement: Supplementary file 2 — Reporting Summary [file 41477_2025_2176_MOESM2_ESM.pdf]

nature portfolio

Corresponding author(s): Rüdiger Simon

Last updated by author(s): Nov 4, 2025

Reporting Summary

Nature Portfolio wishes to improve the reproducibility of the work that we publish. This form provides structure for consistency and transparency in reporting. For further information on Nature Portfolio policies, see our Editorial Policies and the Editorial Policy Checklist.

Statistics

For all statistical analyses, confirm that the following items are present in the figure legend, table legend, main text, or Methods section.

n/a	Confirmed	
☐	☒	The exact sample size (*n*) for each experimental group/condition, given as a discrete number and unit of measurement
☐	☒	A statement on whether measurements were taken from distinct samples or whether the same sample was measured repeatedly
☐	☒	The statistical test(s) used AND whether they are one- or two-sided<br>*Only common tests should be described solely by name; describe more complex techniques in the Methods section.*
☐	☒	A description of all covariates tested
☐	☒	A description of any assumptions or corrections, such as tests of normality and adjustment for multiple comparisons
☐	☒	A full description of the statistical parameters including central tendency (e.g. means) or other basic estimates (e.g. regression coefficient) AND variation (e.g. standard deviation) or associated estimates of uncertainty (e.g. confidence intervals)
☐	☒	For null hypothesis testing, the test statistic (e.g. *F*, *t*, *r*) with confidence intervals, effect sizes, degrees of freedom and *P* value noted<br>*Give P values as exact values whenever suitable.*
☒	☐	For Bayesian analysis, information on the choice of priors and Markov chain Monte Carlo settings
☒	☐	For hierarchical and complex designs, identification of the appropriate level for tests and full reporting of outcomes
☐	☒	Estimates of effect sizes (e.g. Cohen's *d*, Pearson's *r*), indicating how they were calculated

Our web collection on statistics for biologists contains articles on many of the points above.

Software and code

Policy information about availability of computer code

Data collection

Confocal images were acquired by Confocal Microscopy (Zeiss, LSM880) by ZEN software. For Bulk RNA-seq data collection, we used Illumina NextSeq platform (Biomarker Technologies) and Illumina NextSeq2000 for ScRNA-Seq. For multiplex smRNA-FISH, we used the Molecular Cartography platform developed by Resolve Biosciences.

Data analysis

For Bulk RNA-Seq analysis: Salmon (v. 0.14.1) and the R packages: EdgeR (v3.32.1) and ThreeDRNAseq.

For ScRNA-Seq analysis: BD Rhapsody Sequence Analysis Pipeline V2.0; R version 4.3.2 and R packages: Seurat (v4.3.0 and v5.0.3), MAST (1.28.0), ScCustomize (2.1.2), Monocle3 (1.3.7), ggplot2 (3.5.1), ComplexHeatmap (2.18.0) and viridis (0.6.5).

For multiplex smRNA-FISH analysis: the cell segmentation was done by the automated pipeline from Resolve Biosciences, which uses MindaGap, Cellpose, and Baysor. In the transverse section to analyze vascular tissue, the cell segmentation was done manually. Cluster annotation was complemented with Mercator v4.6 for scRNA-seq and smRNA-FISH. Barcodes were determined with PERSIST. Image coordinates were obtained with ImageJ/Fiji v2.9.0/875 1.53t and with a Python script using the pillow 882 library (v9.4.0), scipy (v1.10.1) and scikit-image (v0.19.3).

For BARVISTA: the web application in JavaScript was developed with D3.jsv7.8.4 and dom-to-imagev2.6.0

Code availability: we adapted scripts for our data format input.
For Bulk-RNA seq: https://github.com/cvanges/spike_development. For ScRNA-Seq: https://github.com/satijalab/seurat or Pseudotime analysis: https://github.com/cole-trapnell-lab/monocle3. For multiplex smRNA-FISH cell segmentation: https://github.com/ViriatoII/MindaGap; https://github.com/MouseLand/cellpose; https://github.com/kharchenkolab/Baysor. For barcodes: https://github.com/iancovert/

persist. For data pre-processing: https://imagej.net/ij/, https://python-pillow.org/, https://scipy.org/, https://scikit-image.org/. For web application: https://d3js.org/, https://github.com/tsayen/dom-to-image/tree/master.

For manuscripts utilizing custom algorithms or software that are central to the research but not yet described in published literature, software must be made available to editors and reviewers. We strongly encourage code deposition in a community repository (e.g. GitHub). See the Nature Portfolio guidelines for submitting code & software for further information.

Data

Policy information about availability of data

All manuscripts must include a data availability statement. This statement should provide the following information, where applicable:

- Accession codes, unique identifiers, or web links for publicly available datasets
- A description of any restrictions on data availability
- For clinical datasets or third party data, please ensure that the statement adheres to our policy

Metadata associated with this investigation can be accessed here: http://purl.org/barvista/arc
BARVISTA is accessible here: http://purl.org/barvista/home

Research involving human participants, their data, or biological material

Policy information about studies with human participants or human data. See also policy information about sex, gender (identity/presentation), and sexual orientation and race, ethnicity and racism.

Reporting on sex and gender	NA
Reporting on race, ethnicity, or other socially relevant groupings	NA
Population characteristics	NA
Recruitment	NA
Ethics oversight	NA

Note that full information on the approval of the study protocol must also be provided in the manuscript.

Field-specific reporting

Please select the one below that is the best fit for your research. If you are not sure, read the appropriate sections before making your selection.

☒ Life sciences ☐ Behavioural & social sciences ☐ Ecological, evolutionary & environmental sciences

For a reference copy of the document with all sections, see nature.com/documents/nr-reporting-summary-flat.pdf

Life sciences study design

All studies must disclose on these points even when the disclosure is negative.

Sample size	For Bulk RNA-Seq around 25 spikes were pooled in each replicate. For ScRNA-Seq, in W3.5 over 16500 high-quality cells were considered for data analysis. In W0.5, 910 cells were considered. In com1.a;com2.g, 5123 cells were analyzed. For multiplex smRNA-FISH (Molecular Cartography) around 20000 cells were segmented and analyzed (W3.5).
Data exclusions	For scRNA-seq, low-quality cells (based onUMI counts) were removed for subsequent analysis. Differentially Expressed Genes by the protoplasting process (based on Bulk RNA-seq) were excluded from the dataset. For multiplex smRNA-FISH , 100 probes were designed, but during data integration (ScRNA-Seq with smRNA-FISH) were excluded those with no signal or affected by protoplasting, using 81 (W3.5), 76 (W0.5) and 83 (com1.a;com2.g) as anchor genes.
Replication	For Bulk RNA-seq and ScRNA-Seq were used three independent experiments with around 25 developing spikes each, all collected independently. For multiplex smRNA-FISH, two consecutive sections were analyzed per individual, and around 3 spikes were imaged from at least 9 attached to the slides. For Golden Promise, we have two independent experiments.
Randomization	All plants were growing under the same conditions. In each independent experiment a batch of plants was collected with weeks apart between batch. For mulitplex smRNA-FISH the tissue was collected in two experimental rounds.
Blinding	For ScRNA-Seq: we integrated the data from three independent experiments (W3.5) to avoid bias or batch-effects. Cluster annotation was done using known markers in this integrated reference.

Reporting for specific materials, systems and methods

We require information from authors about some types of materials, experimental systems and methods used in many studies. Here, indicate whether each material, system or method listed is relevant to your study. If you are not sure if a list item applies to your research, read the appropriate section before selecting a response.

Materials & experimental systems

n/a	Involved in the study
☒	☐ Antibodies
☒	☐ Eukaryotic cell lines
☒	☐ Palaeontology and archaeology
☒	☐ Animals and other organisms
☒	☐ Clinical data
☒	☐ Dual use research of concern
☐	☒ Plants

Methods

n/a	Involved in the study
☒	☐ ChIP-seq
☒	☐ Flow cytometry
☒	☐ MRI-based neuroimaging

Plants

Seed stocks	Golden Promise seeds were provided by Prof. Dr. Maria von Korff Schmising (HHU-Dusseldorf, CEPLAS); com1.a;com2.g homozygous double mutant seeds were provided by Prof. Dr. Thorsten Schnurbusch (IPK, Gatersleben); pHvFCP1:mVenus-H2B was generated by our Lab and published in: https://doi.org/10.1038/s41467-025-59330-z
Novel plant genotypes	NA
Authentication	Golden Promise and com1.a;com2.g were self-pollinated and verified by their spike phenotype. pHvFCP1:mVenus-H2B was PCR genotyped.
